# Preparing clinical-grade myeloid dendritic cells by electroporation-mediated transfection of *in vitro *amplified tumor-derived mRNA and safety testing in stage IV malignant melanoma

**DOI:** 10.1186/1479-5876-4-35

**Published:** 2006-08-15

**Authors:** Svetomir N Markovic, Allan B Dietz, Carl W Greiner, Mary L Maas, Greg W Butler, Douglas J Padley, Peggy A Bulur, Jacob B Allred, Edward T Creagan, James N Ingle, Dennis A Gastineau, Stanimir Vuk-Pavlovic

**Affiliations:** 1 Division of Hematology, Department of Internal Medicine, Mayo Clinic College of Medicine, Rochester, Minnesota, USA; 2 Stem Cell Laboratory, Mayo Clinic Cancer Center, Mayo Clinic College of Medicine, Rochester, Minnesota, USA; 3 Human Cellular Therapy Laboratory, Division of Transfusion Medicine, Department of Laboratory Medicine and Pathology, Mayo Clinic College of Medicine, Rochester, Minnesota, USA; 4 Cancer Center Statistics, Mayo Clinic Cancer Center, Mayo Clinic College of Medicine, Rochester, Minnesota, USA; 5 Department of Oncology, Mayo Clinic College of Medicine, Rochester, Minnesota, USA

## Abstract

**Background:**

Dendritic cells (DCs) have been used as vaccines in clinical trials of immunotherapy of cancer and other diseases. Nonetheless, progress towards the use of DCs in the clinic has been slow due in part to the absence of standard methods for DC preparation and exposure to disease-associated antigens. Because different *ex vivo *exposure methods can affect DC phenotype and function differently, we studied whether electroporation-mediated transfection (electrotransfection) of myeloid DCs with *in vitro *expanded RNA isolated from tumor tissue might be feasible as a standard physical method in the preparation of clinical-grade DC vaccines.

**Methods:**

We prepared immature DCs (IDCs) from CD14^+ ^cells isolated from leukapheresis products and extracted total RNA from freshly resected melanoma tissue. We reversely transcribed the RNA while attaching a T7 promoter to the products that we subsequently amplified by PCR. We transcribed the amplified cDNA *in vitro *and introduced the expanded RNA into IDCs by electroporation followed by DC maturation and cryopreservation. Isolated and expanded mRNA was analyzed for the presence of melanoma-associated tumor antigens gp100, tyrosinase or MART1. To test product safety, we injected five million DCs subcutaneously at three-week intervals for up to four injections into six patients suffering from stage IV malignant melanoma.

**Results:**

Three preparations contained all three transcripts, one isolate contained tyrosinase and gp100 and one contained none. Electrotransfection of DCs did not affect viability and phenotype of fresh mature DCs. However, post-thaw viability was lower (69 ± 12 percent) in comparison to non-electroporated cells (82 ± 12 percent; *p *= 0.001). No patient exhibited grade 3 or 4 toxicity upon DC injections.

**Conclusion:**

Standardized preparation of viable clinical-grade DCs transfected with tumor-derived and *in vitro *amplified mRNA is feasible and their administration is safe.

## Background

Dendritic cells (DCs) have been used in numerous recent clinical trials as vaccines intended to break tolerance to tumors and induce tumor-specific therapeutic immunity [1]. To break tolerance to the tumor, DCs must effectively present tumor-associated antigen(s). Antigens have been delivered to DC as whole-tumor lysates, natural or synthetic peptides, recombinant viruses, tumor specific RNA, and recombinant DNA [2]. Most of these sources have been used for clinical trials, particularly tumor lysates; however, tumor lysates are a limited and inconsistent source of antigenic material. The optimal method for antigen delivery to DCs is still controversial [2].

A current discussion of antigen delivery to DCs concluded that "the most constitutive and prolonged MHC I presentation would likely result from processing of endogenously produced proteins located in the cytoplasm; like other cells, DC present self- or virus-derived endogenous antigens generated via proteasome degradation of newly synthesized ubiquitinated proteins" [3]. These requirements limit the choice of antigen delivery to viral constructs, DNA or RNA. Among these options, delivery of tumor-derived RNA has been the most reliable, and efficient as it induced the highest level of immunity [4-6]. Delivery of RNA containing entire protein coding sequences eliminates the need to select patients on the basis of their HLA antigens, a restriction characteristic of the use of antigenic peptides.

In our efforts to contribute to the development of DC-based vaccination strategies for immunotherapy, others and we are studying metastatic malignant melanoma. Metastatic melanoma is an incurable condition with a median survival time of nine months and a less than 5 percent likelihood of survival five years following diagnosis [7] with a continuing increase in age-adjusted mortality [8]. One of the most intriguing investigational approaches to melanoma therapy has been immunotherapy [9]. Driven in part by anecdotal reports of spontaneous resolution of metastases and broad resistance to numerous conventional chemotherapy agents, immunotherapy of melanoma has been the focal point of clinical cancer immunotherapy [10-13]. DC vaccines have been studied for their ability to recruit naïve T cells and stimulate tumor-specific memory T cells for induction of clinical responses [1,14]. Despite some progress, the clinical efficacy of therapeutic DC vaccines remains unpredictable. The absence of significant and predictable clinical responses has been ascribed in part to the lack of a standardized approach to DC preparation, treatment regimens and outcome measurements [2,15].

To develop a standardized method of preparing a clinical-grade myeloid DC vaccine for treatment of malignant melanoma, we explored the use of electroporation-mediated transfection (electrotransfection) as a method amenable to standardization of physical variables. In addition, we employed *ex vivo *amplification of autologous tumor-derived mRNA [16,17] as it allows a level of standardization of the process and final product. Electroporation has been validated in numerous laboratory studies [6,18-21] and has been used for transfection of unexpanded tumor-derived RNA [21]. Here we report our experience in preparing clinical-grade myeloid DCs by electrotransfection of *in vitro *amplified tumor-derived mRNA and safety testing of the DC product in patients suffering from stage IV malignant melanoma.

## Methods

### Dendritic cell vaccine preparation

The overall scheme of DC vaccine preparation included separation of IDCs from autologous CD14-positive cells and isolation of total RNA from autologous tumor tissue. RNA was reversely transcribed to obtain cDNA and amplified using cDNA as template incorporating a T7 RNA promoter. Amplified cDNA was *in vitro *transcribed and loaded into IDCs by electroporation. The DCs were subsequently matured in the presence of inflammatory cytokines and cryopreserved as single aliquots prior to use.

#### Dendritic cells

Mononuclear cell were collected from patients by conventional leukapheresis. The collected cells were incubated with clinical-grade CD14-specific immunomagnetic reagent (Miltenyi Biotec, Auburn, CA) and separated on a CliniMACS apparatus (Miltenyi) using *Enrichment 1.1 *program. The cells were further processed by the previously described method [22,23] in a Class 10,000 cGMP-grade cell processing facility equipped with Class-100 biological safety cabinets for aseptic manipulation. Briefly, CD14^+ ^cells were plated in polystyrene flasks at 2 × 10^6 ^cells/mL in X-VIVO 15 medium (BioWhittaker, Walkersville, MD) containing 1.0 percent pooled human AB serum (HABS; C-6 Diagnostics, Mequon, WI) GM-CSF (800 IU/mL, Immunex, Seattle, WA) and IL-4 (1000 IU/mL, R&D Systems, Minneapolis MN). The flasks were incubated at 37°C in a humidified atmosphere of 5 percent CO_2_. One mL of the same medium, but with GM-CSF increased to 1600 IU/mL, was added per three mL of the medium three and five days later, respectively. On the seventh day non-adherent cells were collected and resuspended in the electroporation medium and electrotransfected with *in vitro *amplified tumor-derived RNA (see below). RNA-loaded DCs were resuspended at 1.0 × 10^6^/mL in X-VIVO 15, 1.0 percent HABS, 800 IU/mL GM-CSF, 1000 IU/mL IL-4, 1100 IU/mL TNF-α, 1870 IU/mL and 1.0 μg/mL prostaglandin E_2 _(PGE_2_). Non-adherent MDCs were collected two days later, tested for sterility, viability, and phenotype (by expression of CD14, CD83 and CD86). DC products were predominantly CD14-negative (12 ± 6 percent of cells were CD14^low^), CD86^high ^(96 ± 2 percent) and mature (80 ± 6 percent CD83^+^), in accord with our earlier experience [23]. For a more detailed characterization, we measured also the expression of HLA-A, B, C, HLA-DR, CD40, CD54, CD80, CD209 and CCR7 in DCs from Patient 1 and Patient 5.

#### Cell characterization by flow cytometry

We characterized the cells by flow cytometry with a FACSCalibur flow cytometer (BD Biosciences, San Jose, CA) and the fluorophore-conjugated monoclonal antibodies with specificity indicated in Table 1. For each analysis we recorded one hundred thousand counts. Data were analyzed with CellQuest software (BD Biosciences).

**Table 1:** Immunoreagents used in this study

**Antibody specificity**	**Fluorescent label**	**Manufacturer**
**HLA-ABC**	PE^a^	BD Pharmingen
**HLA-DR**	FITC	Biosource^b^
**CD14**	PE	eBioscience
**CD40**	FITC	BD Pharmingen
**CD54**	PE	Biosource
**CD80**	FITC	BD Pharmingen
**CD83**	PE	Immunotech^c^
**CD86**	FITC	Ancell ^d^
**CD209**	FITC	eBioscience
**CCR7**	FITC	R&D Systems
**IgG**	PE	Biosource

^a ^FITC, fluorescein isothiocyanate; PE, phycoerythrin; APC, allophycocyanin; ^b ^Camarillo, CA; ^c ^Miami, FL; ^d ^Bayport, MN.

#### Tumor RNA extraction

Tumor tissues were surgically resected, aseptically collected, and immediately transported to the cell processing facility. Fresh tissue was cut into cubes measuring approximately 3 × 3 × 3 mm. Each piece was placed into a separate 1.5 mL RNase-free tube and covered with RLT buffer (Qiagen, Valencia, CA). Tissues were homogenized with the Pellet Pestle™ (Kontes, Vineland, NJ). To each sample 100 μL of RNase-free water and 350 μL of RLT buffer (containing 10 μL β-mercaptoethanol per mL of RLT buffer) were added and the sample was briefly vortexed and quickly centrifuged. Then 250 μL of 96 percent ethanol was added and thoroughly mixed by pipetting. The sample viscosity was reduced using a Qiashredder (Qiagen) and 700 μL was applied onto an RNeasy™ mini column (Qiagen) as directed by the manufacturer. Purified total RNA was collected in 50 μL of RNase-free water. RNA was quantified photometrically at 260 nm using  the extinction coefficient of 0.025 for a 1.0 µg/mL solution and its integrity was monitored by agarose gel electrophoresis and in some cases confirmed by the use of an Agilent Bioanalyzer™ (Agilent Technologies, Palo Alto, CA) at the Mayo Clinic Microarray Core Facility. A sample was acceptable for further use if it contained more than 2.5 μg of RNA.

#### Reverse transcription and RNA amplification

Autologous tumor RNA was amplified and prepared for *in vitro *transcription as described by others [24]. Briefly, 2.5 μg of total tumor RNA per reaction was incubated with the first strand primer (5'-AAGCAGTGGTATCAACGCAGAG TACT_(30)_VN-3'; where V is G, A, or C and N is any nucleotide) and the dNTP mixture at 65°C for 5 min and 8°C for 10 min [24]. We then added DTT, the reaction buffer, RNaseOUT (Invitrogen, Carlsbad, CA) and Superscript II reverse transcriptase (Invitrogen) and reversely transcribed the RNA at 42°C for 35 min. Then 10 pmol of the T7 Switch primer (5'-CTAATACGACTCACTATAGGGCGG G-3') was added and the solution was further incubated at 42°C for 30 min, at 70°C for 15 min and 42°C for 2 min. The resulting cDNA (2 μL per reaction) was used as template for a 100-μL PCR reaction using the Advantage™ polymerase (Clontech, Mountain View, CA) according to the manufacturer's directions with 20 pmol of the T7-5' (5'CCATCCTAATACGACTCACTATAG-GGC-3'), and T7-3' (5'-AAGCAGTGGTATCAACGCAGAGT-3') as primers. PCR included one cycle at 95°C for 1 min; 25 cycles of 95°C for 15 sec, 65°C for 20 sec and 68°C for 6 min; and the final incubation at 68°C for 7 min. All primers were synthesized at the Mayo Clinic DNA Synthesis Core Facility.

To transcribe the RNA *in vitro*, for each reaction we added sequentially the following reagents to a RNase-free tube at room temperature: RNase-free water to bring the final volume to 20 μL, 10 μL 2× NTP/CAP, 2 μL 10× buffer, 1 μg cDNA, and 2 μL mMessage mMachine RNA polymerase mix (Ambion, Austin, TX). Typically, we performed five to 15 reactions per sample. After *in vitro *transcription the individual reaction products were pooled, and excess nucleic acids and primers removed by gel filtration using a NucAway spin column (Ambion) centrifuged at 750 × g for 2 min. The tube containing the bulk of the RNA was frozen and stored at -70°C. Aliquots were analyzed for RNA quantity and integrity by agarose gel electrophoresis and/or capillary microelectrophoresis by use of the Agilent Bioanalyzer 2100.

#### Dendritic cell electrotransfection with RNA

For experiments aimed at optimizing electroporation conditions, a cDNA encoding the enhanced green fluorescent protein (eGFP) gene and containing a T7 promoter and polyadenylation signal suitable for DC transfection was generously provided by S. Sæbøe-Larssen, University of Oslo, Oslo, Norway, and prepared as described in ref. [18]. The plasmid served as template for *in vitro *transcription using mMESSAGE mMACHINE kits (Ambion, Austin, TX). The transcription reaction mixture was purified using Nuc Away spin columns (Ambion) according to manufacturer's instruction. Concentration of the resulting mRNA was measured photometrically at 260 nm and 280 nm. For transfection into DCs, mRNA was dissolved in water at 1.0 mg/mL.

Immature DCs manufactured from the blood of melanoma patients were electrotransfected with mRNA isolated from autologous tumor tissue and *in vitro *amplified [24]. The cells were prepared as above except that the IDCs were washed, suspended in the Cytoporation Formula R Medium (Cyto Pulse Sciences, Inc., Glen Burnie, MD) at a density of 1 × 10^7^/mL in the presence of 20–50 μg/mL of autologous mRNA. The cell suspension was transferred to sterile, disposable electroporation cuvettes with a 4-mm electrode gap (Molecular BioProducts, San Diego, CA). The cells were subjected to two square 400-V pulses of 50 μs each from the PA-4000 PulseAgile generator. Following electroporation the cells were rested in X-VIVO 15 medium containing HABS, GM-CSF and IL-4 as above at 37°C in humidified 5 percent CO_2_ for one hour. Subsequently the cells were washed once and suspended in the maturation medium containing 1100 IU/mL TNF-α and 1.0 μg/mL PGE_2_ for two more days. MDCs were collected, assayed for compliance with release criteria and administered as described above. All vaccines were frozen and thawed prior to administration.

#### Melanoma-specific transcripts in amplified mRNA

Total RNA was extracted from patients' melanoma tissues and from cell lines Sk-mel 28 (HTB-72, American Type Culture Collection, Manassas, VA) and T2 cells (CRL-1992, American Type Culture Collection) as positive and negative control, respectively. RNA samples were tested before and after *in vitro *expansion for the presence of transcripts of tumor-associated molecules gp100, tyrosinase, and Mart1 and of G6PDH (as positive control). Briefly, 1 μg of RNA was used as a template in a 50-μL single-tube RT-PCR reaction using Titan RT-PCR (Roche Diagnostics, Indianapolis, IN), one set of primers, and 30 cycles of PCR according to manufacturer's directions. Primer sequences and predicted amplicon sizes are indicated in Table 2. Products of each reaction were analyzed by electrophoresis on ethidium-bromide-stained 1.0 percent agarose gel and scored for the presence or absence of the transcript.

**Table 2:** Primer sequences and predicted amplicon sizes in detection of melanoma-specific transcripts in dendritic cells

**Transcript**	**Sequence**	**Predicted amplicon size (bp)**	**Ref**.
**GAPDH**	GAA GGT GAA GGT CGG AGT C	226	[29]
**GAPDH**	GAA AGA TGG TGA TGG GAT TC		
**Mart1 Out1**	ATG CCA AGA GAA GAT GCT CAG	384	
**Mart1 Out2-2**	AGC ATG TCT CAG GTG TCT CG		
**gp100 Out1**	GCT TGG TGT CTC AAG GCA ACT	751	[30]
**gp100 Out2-2**	CTC CAG GTA AGT ATG AGT GAC		
**Tyrosinase Out1**	TTG GCA GAT TGT CTG TAG CC	284	
**Tyrosinase Out2-2**	AGG CAT TGT GCA TGC TGC TT		

### Clinical protocol

This trial enrolled six patients who were at least 18 years of age with histologically proven stage IV malignant melanoma. Three patients met the RECIST criteria for measurable disease [25], two had evaluable disease only and one patient had resected metastatic disease and was followed for progression. Contraindications to study entry included: unsatisfactory hematologic or blood chemistry profile (defined as absolute neutrophil count below or equal to 1,500/mL, platelet count below or equal to 100,000/mL, hemoglobin below 9.0 g/dL, serum alkaline phosphatase three times above the institutional upper limit of normal (ULN), aspartate transferase three times above ULN or creatinine 1.5 times above ULN, life expectancy of less than 12 weeks, ECOG performance status of 3 or 4, uncontrolled infection, prior immunization with differentiation antigen peptides, recent chemotherapy/immunotherapy/radiation therapy (less than one month prior to registration), known central nervous system metastases or carcinomatous meningitis, seizure disorder, active psychiatric disorder requiring pharmacologic therapy, known immune deficiency, history of other malignancies within the last five years, and inability to provide informed written consent. Women of childbearing potential were required to have a serum pregnancy test at most seven days prior to registration. Women of childbearing age who were unwilling to employ adequate contraception, pregnant women, and women who were nursing were not eligible.

Following registration, patients underwent surgical resection of a symptomatic tumor mass followed by a single leukapheresis on average 29 days later (range: 18 to 40 days). Upon generation of the DC vaccine and recovery from surgery, patients underwent a series of four subcutaneous injections of the DC vaccine (5 × 10^6 ^cells/treatment) administered once every three weeks.

Intra-patient dose modifications were not allowed. If at the time of re-treatment a patient had a grade 3 or 4 non-hematologic toxicity or grade 2 bronchospasm, generalized allergic reaction or autoimmune reaction, all further study treatments were discontinued.

Prior to each cycle of treatment and at the time of progression, patients underwent a physical examination, toxicity assessment using the NCI common toxicity criteria [26], evaluation of injection site skin reaction, and complete blood count and serum chemistry profiling. Tumors were to be assessed according to RECIST criteria at weeks 6 and 12 after treatment initiation and every three months thereafter for up to two years. In this HLA-unrestricted study, measurements of T cell function included vaccine-stimulated proliferation *in vitro *and levels of intracellular and secreted IFN-γ during and after treatment.

### Clinical study end-points

The principal end-point of this study included the determination of the safety and toxicity profile of the mRNA-transfected autologous DC vaccine administered to patients with stage IV melanoma. Although the clinical status of the patients made the expectations for major immune and/or clinical effects of treatment unlikely, our secondary end-points included a description of immunization efficacy of the vaccine and the collection of preliminary descriptive data of clinical efficacy (tumor responses, progression free survival and overall survival) of the DC vaccine.

Six patients were accrued to the study. Enrollment was to be suspended if two or more of the six patients experienced a grade 4 hematologic toxicity lasting five or more days or a rise in serum creatinine of two or more times above the pretreatment value. All patients who fulfilled the eligibility criteria and received one injection were included in all analyses. Tumor response rate was estimated on the number of eligible patients who achieved a complete or partial remission on two consecutive evaluations divided by the total number of eligible patients enrolled.

### Immune response monitoring

Peripheral blood was drawn before the first DC vaccination, after the third vaccination and one month after the fourth and final vaccination when possible. We isolated T cells by negative immunomagnetic adsorption (Miltenyi) and cryopreserved them until they could be analyzed in a single experiment after the completion of the trial. In addition, we compared the mRNA-transfected DCs with DCs that were not electroporated for the ability to stimulate proliferation of and IFN-γ secretion by T cells.

#### Measuring IFN-γ secretion by ELISpot

We coated 96-well plates (Multiscreen; Millipore, Bedford, MA) with the capture monoclonal antibody specific for human IFN-γ (1:500 dilution, eBioscience, San Diego, CA) overnight at 4°C. Plates were further incubated with blocking solution (X-VIVO 15 supplemented with 10 percent Human AB serum and 1.0 percent penicillin/streptomycin solution), 200 μL per well, for two hours at room temperature. T cells were seeded in quadruplicate wells in a tenfold excess over mRNA-transfected or mock-transfected DCs in X-VIVO 15 supplemented with 1.0 percent human AB serum and 1.0 percent penicillin/streptomycin. Alternatively, the DCs were incubated with Fluzone^® ^influenza virus vaccine (2005–2006 formula; Aventis Pasteur, Swiftwater, PA), 5.0 μL/mL, for two hours and washed prior to plating. Cell cultures were incubated in a humidified incubator with 5 percent CO_2 _at 37°C for 48 hours. Plates were incubated with the biotinylated IFN-γ-detection antibody (1:500 dilution; eBioscience) for two hours at room temperature, washed, and incubated for one hour with avidin-HRP (eBioscience), diluted 1:1000 in PBS containing 1% Human AB serum and washed. Then 100 μL of substrate 3-amino-9-ethylcarbazole (AEC) solution (BD Biosciences, San Diego, CA) was added per well. Spots were allowed to develop for 30 minutes, washed with water and enumerated by an ImmunoSpot plate reader (Cellular Technology, Ltd., Cleveland, OH).

#### Mixed lymphocyte reaction

We evaluated the ability of MDCs to stimulate T cell proliferation and the proliferation capacity of T cells by mixed lymphocyte reaction (MLR). MDCs from melanoma patients and four normal subjects were added each at 1.0 × 10^4 ^per well in 96-well plates containing X-VIVO 15 medium supplemented with 1.0 percent HABS and 1.0 percent penicillin/streptomycin. One hundred thousand T cells mixed from four healthy donors in equal proportions were added to each well containing the MDCs in a final volume of 200 mL. The cells were co-incubated for 84 hours. Twelve hours prior to cell collection with a Skatron (Sterling, VA) semiautomatic cell harvester, [^3^H]-thymidine (1.0 μCi in 100 μL) was added to each well. Radioactivity incorporated into DNA was measured by a LS 6000SC (Beckman-Coulter, Fullerton, CA) scintillation counter. To evaluate the capacity of individual T cells from the patients and healthy controls, we followed the same procedure except that we used a mixture of equal proportions of MDCs from four healthy donors.

### Statistical analysis

Differences among characteristics of DC data groups were assessed by the two-tailed *t*-test for independent samples with equal variance. All data sets contained measurements from each of the six patients (*n *= 6). In all analyses we used Prism 4.0 software (GraphPad, San Diego, CA) for Macintosh computers.

## Results

### Feasibility of preparing autologous tumor-mRNA transfected DC vaccines

The primary laboratory goal of this study was to evaluate the feasibility of constructing a clinical grade autologous cancer vaccine using autologous DCs transfected with *in vitro *amplified tumor mRNA. RNA was extracted and amplified as specified above. Pertinent data documenting the efficiency of cDNA amplification and *in vitro *transcription are shown in Table 3. The total amplification factor (*e.g*., how many times RNA isolated from the tumor has been amplified before used for transfection) is calculated as the product of the PCR amplification factor and the transcription amplification factor. Total masses and volumes are those entered into the respective amplification step, not the total amount isolated from the tumor.

**Table 3:** Isolated total RNA mass and volume, mass of cDNA after PCR amplification and the corresponding data for RNA amplified by *in vitro *transcription together with amplification factors for each step and overall amplification

**Pt No**.	**Total RNA volume (μL)**	**Total RNA mass (μg)**	**Amplified cDNA (μg)**	**PCR amplification factor**	**Amplified RNA volume (μL)**	**[Amplified RNA] (μg/μL)**	**Transcription amplification factor**	**Overall amplification factor**^**a**^
**1**	40	15.7	63.5	15.9	400	31.1	195	3100
**2**	90	30.4	74.0	18.5	1000	2.1	27	500
**3**	100	10.4	51.9	9.9	2013	1.8	70	700
**4**	35	15.8	75.1	10.7	1471	2.2	44	500
**5**	155	78.9	63.2	9.0	1320	5.2	109	1000
**6**	40	20.2	99.0	14.1	1592	1.7	37	500
**Avg**	76.67	28.6	71.1	13.0	1299	7.4	81	1100
**SD**	47.75	25.5	16.1	3.8	552	11.7	63	--

^a^Rounded to the nearest hundred.

**Figure 1:**
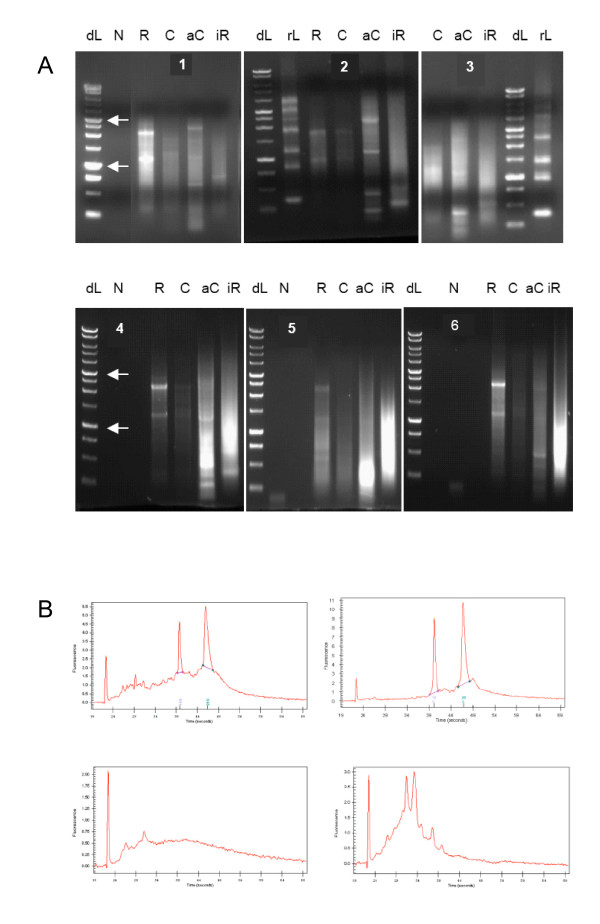
**(A) Size distribution of nucleic acids obtained during processing**. Nondenaturing agarose gel electropherograms showing the size distribution of total RNA extracted from the tumor (R), cDNA after reverse transcription (C), amplified cDNA (aC) and *in vitro *transcribed RNA (iR). rL stands for RNA marker ladder (gel 3) and N for negative control. Upper arrow at the DNA marker ladder (dL) points to the band of 3 kb and the lower arrow to the band of 1 kb. Numbers refer to patients 1 to 6. **(B) Capillary microelectropherograms of total RNA isolated from tumor tissue (upper row) and the corresponding *in vitro *transcribed RNA (lower row)**. RNA samples from patient 2 are represented on the left and samples from patient 6 on the right. The sharp peak at the left of each plot is a loading control. The two other prominent peaks in the upper plots represent ribosomal RNA.

For all patients we monitored cDNA amplification and *in vitro *transcription by gel electrophoresis (Figure 1A) and for patients 2 and 6 by capillary microelectrophoresis (Figure 1B). We found that the total RNA extracted from tumors consistently displayed the characteristic RNA, ribosomal RNA and cDNA species up to 5 kb in length throughout the entire process (Figure 1A, B). Furthermore, in total RNA and *in vitro *transcribed RNA we monitored the presence of melanoma-specific transcripts gp100, tyrosinase, and MART1 by RT-PCR (Table 4). In only one *in vitro *amplified sample we could not detect a transcript that was present in the total RNA. Thus, the process had sufficient fidelity to maintain the presence of the monitored melanoma-specific transcript throughout *in vitro *amplification.

**Table 4:** Melanoma markers present (+) or absent (-) in native tumor RNA and *in vitro *amplified RNA from patients and from cell lines Sk-Mel (positive control) and T2 (negative control)

**Name**	**GAPDH**	**gp100**	**Tyrosinase**	**MART1**
**Sk-Mel**	+	+	+	+
**T2**	+	a	-	-
**Patient 1**	+/+	+/+	+/+	+/+
**Patient 2**	+/+	+/+	+/+	+/+
**Patient 3**	+/+	-/-	-/-	-/-
**Patient 4**	+/+	+/+	+/+	+/+
**Patient 5**	+/+	+/±	+/+	-/-
**Patient 6**	Not determined			

^a ^Indeterminate

### Optimization of DC electrotransfection with *in vitro *amplified mRNA

We optimized electrotransfection by monitoring transfection efficiency and DC viability as a function of electrode separation, pulse amplitude and length and amplified mRNA concentration in the medium. In all experiments we employed a PA-4000 PulseAgile square-wave generator and the proprietary cGMP-grade low-conductivity (80 μS/cm) Cytoporation Medium Formula R medium (both Cyto Pulse Sciences, Glen Burnie, MD). We transfected IDCs with mRNA encoding the eGFP gene, matured the cells for 48 hours and measured transfection efficiency (by eGFP fluorescence) and viability (by exclusion of 7-amino-actinomycin D, 7-AAD; Pharmingen, San Diego, CA) following transfection in electroporation cuvettes with a 1-mm electrode separation (100 μL volume) and a 4-mm electrode separation (400 μL). With the pulse sequence consisting of two rectangular 1.0-kV/cm 50-μs pulses separated by 500 ms in all experiments (as recommended by the manufacturer), we found no difference in transfection efficiency and post-electrotransfection viability (both being above 90 percent in both cuvettes). Thus, for higher throughput we used the larger cell throughout the study.

**Figure 2:**
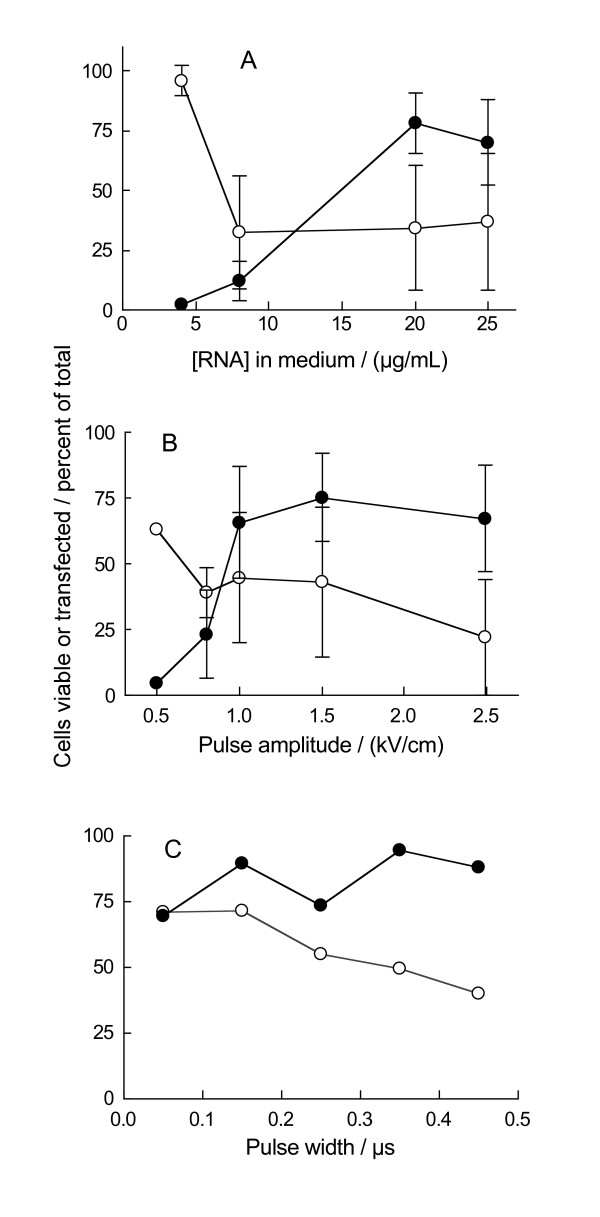
**Identifying conditions for electrotransfection of immature dendritic cells**. Normal IDCs were electrotransfected with *in vitro *transcribed eGFP-mRNA. Following electrotransfection, the cells were matured for 48 hours when viability (open symbols) and transfection efficiency (closed symbols) were quantified (by 7-AAD exclusion and eGFP fluorescence, respectively). Shown are the data from the final iteration in the analysis where mRNA concentration varied from 4.0 μg/mL to 25 μg/mL (A), pulse amplitude from 0.5 kV/cm to 2.5 kV/cm (B) and pulse width from 0.05 μs to 0.45 μs (C). Symbols denote mean values of measurements in cells from three or more individuals ± standard deviation (except in panel C that is an example of an entire experiment conducted with cells from one individual).

To refine the electrotransfection conditions further, we iteratively measured the transfection efficiency and DC viability under different parameter combinations. In the final iteration we varied the mRNA concentration between 4.0 μg/mL and 25 μg/mL, pulse amplitude between 0.5 kV/cm and 2.5 kV/cm and pulse width between 0.05 μs and 0.45 μs. From the dependence of transfection efficiency and viability on mRNA concentration (Figure 2A), we optimized the effect of pulse amplitude for mRNA concentration in the 20–25 μg/mL range (Figure 2B). Because the pulse of 1.0 kV/cm resulted in acceptable transfection efficiency and reasonable viability, we further studied the effect of pulse width at 20–25 μg/mL RNA and 1.0 kV/cm (Figure 2C). Based on these data, we selected 1.0-kV/cm 150-μs pulses and 10 μg amplified RNA per one million cells as an optimal compromise of parameters for DC manufacturing.

### Electrotransfected DCs translate exogenous mRNA

**Figure 3:**
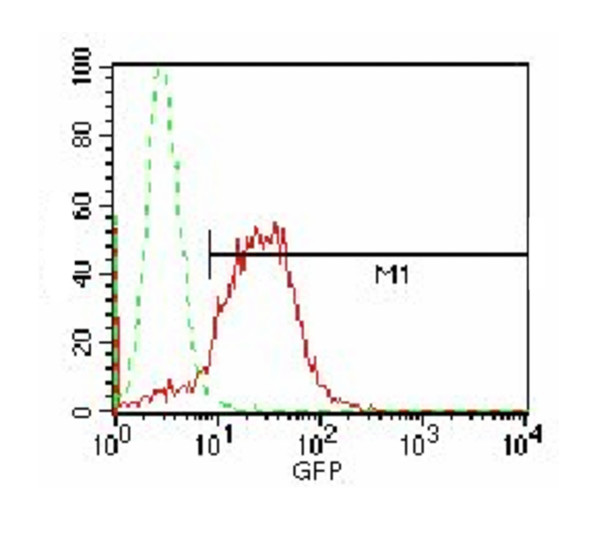
**Electrotransfected dendritic cells can express transfected mRNA**. IDCs, 1.0 × 10^7 ^cells/mL, were electroporated by two 1.0-kV/cm 50-μs pulses separated by 500 ms in the presence of 20 μg eGFP-mRNA per 1.0 × 10^6 ^cells, matured for three days when fluorescence due to eGFP was quantified by flow cytometry. Dotted line: control cells electroporated without mRNA; full line, the cells electroporated in the presence of eGFP-mRNA. Shown is a histogram typical of four experiments.

Determination if electrotransfected DCs can translate exogenous transcripts may be complicated by low levels of a transcript in the total mRNA, low levels of the resulting protein, high DC capacity for protein degradation and by the consequent need for very sensitive detection techniques. To mitigate these problems, we monitored expression of the transfected eGFP-mRNA; GFP is uniquely stable within cells (cf. ref. [27]) and its presence can be detected by fluorescence. We transfected IDCs, 1.0 × 10^7 ^cells/mL, in the presence of 20 μg eGFP-mRNA per 1.0 × 10^7 ^cells. Following electrotransfection by two 1.0-kV/cm 50-μs pulses separated by 500 ms, we matured the cells for three days and measured eGFP fluorescence. While control cells (electroporated without mRNA) showed only background fluorescence, the cells electrotransfected with eGFP-mRNA fluoresced due to the presence of the eGFP protein (Figure 3). Thus, DCs can translate exogenous electrotransfected mRNA.

### Dendritic cell characteristics during manufacturing

The percentage of CD14^+ ^cells isolated from apheresis products, purity of isolated CD14^+ ^cells, efficiency of CD14^+ ^cell selection as well as viability and yield of IDCs were indistinguishable between melanoma patients and normal volunteer blood donors used as control [23]. However, the percentage of CD83^+ ^DCs matured from melanoma-patient derived cells was lower than in identically treated cells derived from patients suffering from chronic myelogenous leukemia [23]. The relative paucity of CD83^+ ^melanoma-patient derived DCs was not due to electrotransfection, as the percentage of electrotransfected CD83^+ ^DCs did not differ from untreated controls (*p *= 0.63).

**Figure 4:**
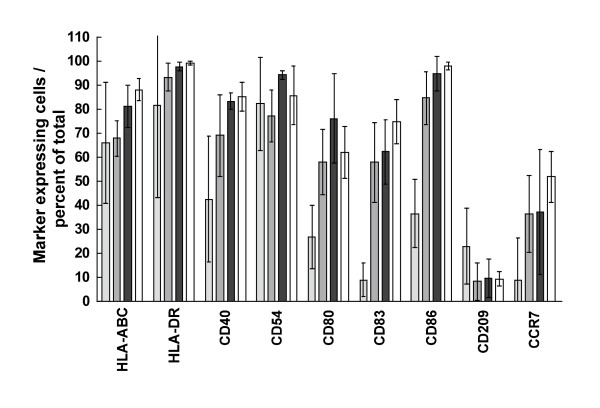
**Expression of surface molecules by patients' dendritic cells**. Flow cytometric characterization of pertinent membrane molecules expressed by normal MDCs (*n *= 4; open columns) and cells from melanoma patients (*n *= 6): IDCs (light shade), MDCs (medium shade) and electrotransfected MDCs (dark shade; for CD83 and CD86, *n *= 6; other molecules were measured in the cells from Patient 1 and Patient 5 only). Shown are mean values ± standard deviation.

Figure 4 summarizes the data for HLA-A, B, C, HLA-DC, CD40, CD54, CD80, CDCD83, CD86 CD209 and CCR7 molecule expression by normal MDCs and native and electrotransfected MDCs from melanoma patients. These data show that patients' MDCs were similar to normal MDCs and that mRNA electrotransfection did not affect MDC membrane molecule expression to any major extent.

While electrotransfection did not affect membrane molecule expression, they did reduce the yield of MDCs; no more than 28.6 ± 16.1 percent of IDCs were recovered as MDCs (in comparison to 70.1 ± 10.6 percent of normal, not electroporated cells) reducing the DC recovery from CD14^+ ^cells to 9.8 ± 3.7 percent (in comparison to 22.9 ± 6.7 percent of normal cells; *p *= 0.003 for both). Immediately after electrotransfection, viability did not differ from normal cells (91.2 ± 4.9 percent *v*. 95.6 ± 4.5 percent), but after 24 hours viability was reduced. In addition, the procedure reduced the post-thaw viability of the cryopreserved product in comparison to nonelectroporated controls (69 ± 12 percent *v*. 82 ± 12 percent, *p *= 0.011).

### Clinical end-points

The clinical protocol accrued a total of six patients with stage IV melanoma (Table 5). Following debulking surgery, the tumor was measurable in three patients, evaluable in two and completely resected in one patient. Prior to DC therapy two patients were treated by radiation and three with interleukin 2.

**Table 5:** Patient characteristics

**Pt No**.	**Age**	**Gender**	**Disease stage**
**1**	52	F	M1a
**2**	52	F	M1a
**3**	66	M	M1a
**4**	49	M	M1a
**5**	40	M	M1c
**6**	63	F	M1c

**Figure 5:**
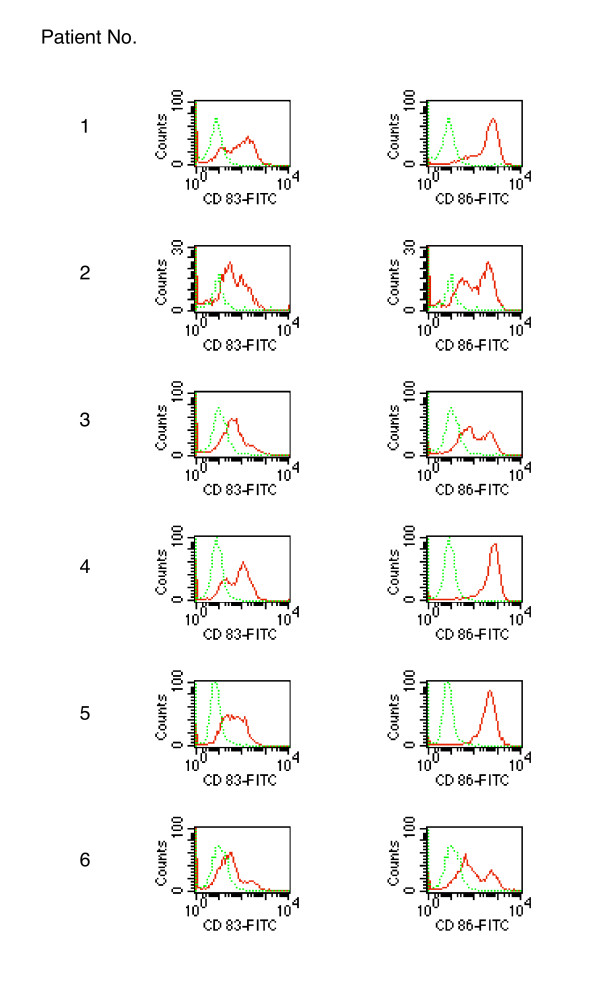
**Expression of CD83 (left) and CD86 (right) by patients' RNA-transfected DCs (red) used for vaccination**. Isotype controls are shown in green.

The primary clinical goal of this phase 1 study was to evaluate the feasibility, safety and toxicity of an autologous MDC vaccine, electrotransfected with *in vitro *amplified autologous tumor-derived genomic mRNA, injected into patients with metastatic melanoma. Figure 5 shows the CD83 and CD86 expression histograms measured in RNA-transfected DCs used for vaccination as part of individual batch release data. The vaccine was safe and non-toxic (no grade 3 or 4 toxicity) with a number of minor (grade 1 and 2) side effects summarized in Table 6.

**Table 6:** Summary of maximum adverse events during dendritic cell treatment and follow-up

**Adverse event**	**Grade 1**	**Grade 2**
Fatigue	1 (1)	1 (1)
Nausea	1 (2)	1 (1)
Anorexia		1 (1)
Arthralgia	1 (1)	
Confusion	1 (1)	
Diarrhea, colostomy	1 (1)	
Diarrhea, no colostomy	1 (2)	
Hemorrhage	1 (2)	
Injection site Rxn	1 (1)	
Myalgia		1 (1)
Pain-abdominal		1 (2)
Pain-bone	1 (1)	
Speech		1 (1)
Vomiting	1 (1)	
Wound, infectious	1 (1)	

Numbers in parentheses indicate the cycle of vaccination when the adverse event occurred. No grade 3 or Grade 4 adverse reactions were observed.

A secondary endpoint of this study was the description of clinical efficacy outcomes in the treated patients. None of the five patients with measurable or evaluable disease experienced an objective clinical response. Of these five patients, one progressed prior to first vaccine injection, one patient progressed at the second injection, two patients progressed at the third injection and one patient remained on study with stable disease. The single patient without assessable disease progressed at the third injection.

**Figure 6:**
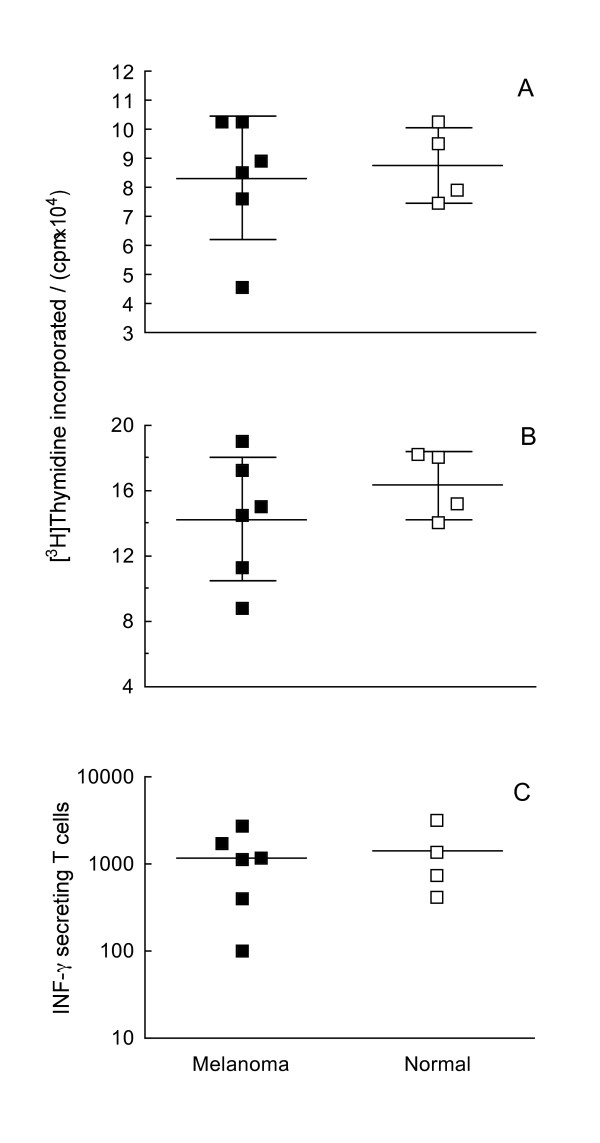
**Functional potential of patients' dendritic cells and T cells**. (**A**) Proliferation of allogeneic T cells (pooled in equal amounts from four healthy donors) stimulated by MDCs from Patient 1–6 (full symbols) and normal MDCs (open symbols); (**B**) Proliferation of patients' T cells (full symbols) and normal T cells (open symbols) in response to stimulation by normal allogeneic MDCs pooled in equal amounts from four normal donors; (**C**) Interferon-γ secretion by patients' T cells (full symbols) and normal T cells (open symbols) upon stimulation by autologous MDCs previously pulsed with an influenza vaccine *in vitro*. Horizontal lines stand for mean values (longer lines) ± standard deviation.

To assess the functional potential of patients' immune cells, we measured the ability of their *ex vivo *matured MDCs to stimulate normal allogeneic T cells *in vitro *and the *in vitro *responsiveness of their T cells to stimulation by normal allogeneic MDCs. In preliminary experiments we compared the ability of normal MDCs electrotransfected with amplified mRNA and native (not electroporated) MDCs to stimulate allogeneic MLR; we found that electrotransfection did not affect the ability of the cells to stimulate proliferation of allogeneic T cells (data not shown). Similarly, patients' MDCs stimulated the proliferation of allogeneic T cells as effectively as normal MDCs (*p *= 0.72; Figure 6A) and patients' T cells responded to stimulation by normal allogeneic MDCs as effectively as normal T cells (*p *= 0.35; Figure 6B). In addition, patients' DCs pulsed *in vitro *with an influenza vaccine were as effective in stimulating autologous T cells to secrete interferon-γ as were the corresponding normal DC/T-cell combinations (*p *= 0.75; Figure 6C). Thus, MDCs and T cells isolated from stage IV melanoma patients can mature and respond to stimulation similarly to normal cells.

We monitored the response of T cells, isolated from patients' blood at different times during treatment, for response to immunization. We measured the proliferation of and interferon-γ release by patients' T cells upon introduction of DCs used for immunization but in no case did we observe any immune response that could be attributed to DC therapy.

## Discussion

Immunotherapy holds the promise of contributing to the limited treatment options available to patients suffering from metastatic melanoma. For active immunization against the disease, DCs are considered advantageous because of their ability to stimulate naïve T cells as well as the memory cells [28]. In addition, the use of whole tumor cells, cell lysates, cell fractions and transcripts as antigens in "education" of DCs for presentation of tumor-associated antigens allows clinical studies without the need for patient selection based on their HLA make-up as is the case in the studies employing chemically defined epitopes (*cf*. refs. 1, 2 and references therein).

We conducted the present study to determine whether mRNA extraction, *in vitro *expansion and electrotransfection into DCs are feasible in the setting of a clinical trial and safe for patients. While previously we reported a preliminary characterization of DC phenotype and viability [23], here we demonstrate that pertinent transcripts of tumor-associated antigens can be detected following transfection of *ex vivo *expanded mRNA into DCs. Thus, this method for preparing clinical-grade DCs is feasible and the resulting cellular vaccine is safe. That we observed neither clinical effects nor immune effects of DCs treatment may not be surprising in view of the severity of disease, limited number of vaccinations and short life expectancy of the patients. Namely, only one patient lived long enough to have received the entire course of therapy while three patients received just two courses of vaccination. Clearly, the next phase of the clinical study will have to be conducted with patients harboring lower tumor burdens and overall in better health, in line with the prevailing thinking that immunotherapy might be most effective within the setting of minimal residual disease.

Recently, Kyte and colleagues evaluated a clinical scale and clinical grade procedure for preparation of DCs transfected with *unamplified *native melanoma-derived mRNA [21]. Their exemplary study allows a comparison of the effects of the transfected native mRNA and amplified mRNA on myeloid DCs. Distribution of molecular sizes in the total RNA isolated in this study (Figure 1B) is similar to the results of Kyte *et al*. [21] who purified mRNA on poly-T beads with the result of enriching the fraction of lower molecular weight mRNA relative to total RNA. We obtained a similar enrichment of lower molecular weight mRNA by *in vitro *expansion. Thus, it appears that both methods yielded qualitatively similar products. Consequently, the less demanding poly-T mediated isolation of native mRNA is advantageous when amounts and quality of the tumor tissue, *i.e*., total RNA, are adequate. On the other hand, the more demanding *in vitro *expansion may be advantageous when the availability of tumor tissue is limited and/or when larger amounts of RNA are needed. Although we demonstrated that the transcripts of antigens of interest are maintained throughout expansion, the complexity of this technically demanding procedure increases the possibility of failure.

DCs transfected with amplified mRNA retain similarly high viability as non-electroporated cells [23] and cells transfected with defined plasmids (*e.g*., for green fluorescent protein and proteinase 3; results not shown) or purified mRNA [21]. However, upon freezing and thawing, DCs transfected with amplified mRNA appeared less viable. This effect does not appear to be caused by electrotransfection alone but may be facilitated by the effects of advanced melanoma on DC precursors. Elucidation of the reasons for the reduced recovery of thawed cells requires further study, but it is possible that some *in vitro *amplified RNA species interfered with recovery. Whatever the cause, reduced cell recovery and viability limit DC manufacturing for clinical trials in advanced melanoma. A possible solution may lie in the recent demonstration that electrotransfecting *mature *DCs results in high yields of viable and functional DCs [6].

In conclusion, tumor-specific vaccination with DCs transfected with *in vitro *amplified tumor-borne mRNA is technically feasible. In the first clinical trial utilizing this technology, we have shown that such immunization is safe. The inherent advantage of this and similar methods is that they are likely to vaccinate against the broad spectrum of tumor-borne antigens that may reduce the ability of tumors to escape immunity by virtue of their genetic instability.
